# Assessment of potential impacts associated with gene flow from transgenic hybrids to Mexican maize landraces

**DOI:** 10.1007/s11248-019-00160-3

**Published:** 2019-06-27

**Authors:** Bill Duncan, Elisa Leyva-Guerrero, Todd Werk, Duška Stojšin, Baltazar M. Baltazar, Silverio García-Lara, Mariana Zavala-López, Juan Manuel de la Fuente-Martínez, Chen Meng

**Affiliations:** 1 Bayer Company, 700 Chesterfield Pkwy. West, St. Louis, MO 63017 USA; 2 Tecnológico de Monterrey, Escuela de Ingeniería y Ciencias, Monterrey, Mexico; 3 Bayer Company, Park Plaza Torre II, 504 Javier Barros Sierra Ave., Col. Santa Fe, Del. Alvaro Obregon, CP 01210 Mexico, DF Mexico

**Keywords:** Maize landraces, Gene flow, GM traits, VT3Pro maize

## Abstract

Genetically modified (GM) maize has been grown and safely consumed on a global scale since its commercialization in 1996. However, questions have been raised about the potential impact that GM maize could have on native maize landraces in Mexico, which is the center of origin and diversity of maize. This research was conducted to evaluate potential changes to maize landraces in an unlikely event of transgene introgression. For this study, two GM traits that confer insect protection and herbicide tolerance in maize (MON 89034 and MON 88017), designated as VT3Pro, were introgressed into two Mexican landraces, Tuxpeño and Tabloncillo. Field trials were conducted across four environments to assess phenotypic characteristics, plant response to stressors, and kernel composition of landraces with and without VT3Pro traits. Furthermore, materials from four backcrossing generations were analyzed for segregation of these GM traits. Generally, no significant differences were observed between landraces with and without VT3Pro traits for the evaluated characteristics and the segregation analysis showed that GM traits, when introgressed into landraces, followed Mendelian principles. These results support the conclusion that, if inadvertently introgressed into landraces, VT3Pro traits are not expected to alter phenotypic or kernel characteristics, plant response to stressors (except for targeted insect protection and herbicide tolerance traits) and would segregate like any endogenous gene. These results should be taken into consideration when discussing benefits and risks associated with commercial production of GM maize hybrids in the centers of origin and diversity of maize.

**Electronic supplementary material:**

The online version of this article (10.1007/s11248-019-00160-3) contains supplementary material, which is available to authorized users.

## Introduction

Maize is an important crop grown over geographically diverse regions, climates, and soil types (Shiferaw et al. 2011). The global production of maize surpasses production of any other crop, having increased 5-fold over the past 56 years (FAOSTAT 2016). This remarkable increase in maize productivity is due to advances in agronomic practices, as well as advances in maize genetics like replacement of open-pollinated populations with maize hybrids and the introduction of biotechnology traits (Troyer 2006).

In 1996, genetically modified (GM) maize became commercially available in the United States. Since then, farmers around the world have rapidly adopted GM maize. In 2015, GM maize constituted a large portion of maize planted in the USA (92%), Brazil (88%), and Argentina (78%) with a total of 60.6 million hectares grown worldwide (ISAAA 2016). Almost all GM maize planted globally contains insect protection and herbicide tolerance traits, either as single transgenes or stacked combination (ISAAA 2016). These and other GM traits have contributed to delivering higher yield, reducing chemical pesticide use, and increasing profitability (Brookes and Barfoot 2018a, b).

The food, feed, and environmental safety of GM maize, as well as their benefits to society, have been demonstrated by global cultivation and consumption of GM maize for over two decades and by studies that showed phenotypic, ecological, and compositional equivalence of GM and conventional crops (Corrales et al. 2018; Phipps and Park 2002; Horak et al. 2007; Drury et al. 2008; Harrigan et al. 2009; Ridley et al. 2011; Sammons et al. 2014; Nakai et al. 2015; Horak et al. 2015a, b; ISAAA 2016; Heredia-Diaz et al. 2016). However, in spite of food, feed, and environmental safety record of GM crops, there remains some hesitation to accepting biotechnology products (ISAAA 2016) and questions have been raised regarding the potential impact that commercial cultivation of GM crops might have in regions where native landraces are traditionally grown by farmers.

Mexico is the center of origin and genetic diversity of maize (Matsuoka et al. 2002). Here, maize remains an important staple crop with economic and cultural value (Espinosa et al. 2003), covering 28% of the total agricultural area (SAGARPA 2017) and yielding on average 3.7 t/ha (FAOSTAT 2016). To meet the national demand, a third of maize consumed in Mexico, totaling over 14 million tons in 2015, comes from import (FIRA 2016).

Of the 7.5 million hectares of maize grown annually in Mexico (FAOSTAT 2016), approximately two-thirds are planted with open-pollinated landraces (Espinosa et al. 2003), which are dynamic populations that are genetically diverse, locally adapted, and have distinct identities. Maize landraces have been selected and maintained via traditional farming systems that, on one hand, maintain the attributes deemed valuable by farmers, like ear and kernel characteristics (Louette and Smale 1998), but on the other hand allow for gene transfer from other landraces, improved maize varieties, or even teosinte (Castillo Gonzalez and Goodman 1995; Louette and Smale 1998; Baltazar et al. 2005; Wang et al. 2017).

A total of 59 races of Mexican maize have been identified based on their molecular and morphological characteristics (Sanchez et al. 2000). Two of the more important landraces, Tuxpeño and Tabloncillo (Sanchez et al. 2000), were evaluated in this study. Landraces of the Tuxpeño type originated from tropical regions of low to medium elevation (Sanchez et al. 2000) and have been widely grown across different states such as Oaxaca, Baja California, Chiapas, Sinaloa, Chihuahua, Sonora, Colima, Durango, Nayarit, Nuevo Leon, Tamaulipas, Hidalgo, Jalisco, Morelos, Puebla, Chiapas, and Yucatan (Aragon-Cuevas et al. 2005; Brush 1995; Louette and Smale 1998; Ortega Paczka 1973; Pineda-Hidalgo et al. 2014; Vazquez-Carrillo et al. 2017; Lazos and Chauvet 2012). The Tuxpeño type landraces are characterized by having cylindrical ears with 12-16 rows of kernels (Sanchez et al. 2000), shorter plant stature, higher yield (Brush 1995), later flowering, large number of tassel branches, white kernels (Baltazar et al. 2005), smaller seed size, and high crude fiber content in grain (Vazquez-Carrillo et al. 2017). Even though their main usage has been for tortilla making, because of their low price and wide availability, they have also been used for specialty products such as pozoles (Vazquez-Carrillo et al. 2017), elotes, botanas, or atoles (Fernandez-Suarez et al. 2013). Furthermore, Tuxpeño type landraces have been used in breeding programs, not only in Mexico, but worldwide (Sanchez et al. 2000).

The Tabloncillo type landraces originated in western Mexico at mid elevations and along the Pacific coastal plains of northwestern Mexico (Sanchez et al. 2000) and have been grown in different states such as Sonora, Jalisco, Sinaloa, Baja California, Chihuahua, Colima, Nayarit, and Guanajuato (Vazquez-Carrillo et al. 2017; Louette and Smale 1998; Pineda-Hidalgo et al. 2014; Lazos and Chauvet 2012). Tabloncillo landraces are characterized by shorter plant stature, earlier maturity, predominantly white kernels (Louette and Smale 1998), longer ears with 8–10 rows of kernels (Sanchez et al. 2000), earlier flowering, fewer tassel branches (Baltazar et al. 2005), larger kernels, low grain expansion volume, high grain protein, and high ash content in grain (Vazquez-Carrillo et al. 2017). They have been typically used for specialty food products like pozoles (Sanchez et al. 2000; Vazquez-Carrillo et al. 2017), pinoles, elotes, botanas, or piznates, but can be used for tortilla making as well (Fernandez-Suarez et al. 2013).

Mexican maize landraces are genetically dynamic populations as they are typically grown in proximity to other landraces or improved varieties to increase cross-pollination among them and allow for selection based on farmer’s preference and use (Louette and Smale 1998; Castillo-Gonzalez and Goodman 1995). This style of traditional farming, a process called “creolization”, allows for the influx of genes into landraces and often includes incorporation of favorable characteristics mostly from improved varieties (Bellon and Berthaud 2004). For example, it was estimated that one third of Tuxpeño landraces showed introgression of genes from improved hybrid varieties (Ortega-Paczka 1973). For the most part, this intentional incorporation of genes from improved varieties has not lead to a dramatic displacement of landraces but resulted in the coexistence of improved varieties and Mexican landraces (Bellon and Berthaud 2004).

In contrast to substantial incorporation of genes from conventional, improved varieties into landraces, there has been evidence of no or limited presence of transgenes into local landraces via seed mixtures or gene flow (Ortiz-Garcia et al. 2005). All indications suggest that landraces are being replaced to a greater extent by industrialization and urbanization of arable land, rather than by modern maize varieties (Chambers et al. 2007).

Because of the unique genetics and cultural importance of landraces in the center of origin of maize, this study was conducted to evaluate the impact that introgression of GM traits into maize landraces may have on their phenotypic and kernel compositional characteristics, responses to stressors, and transgene segregation within a landrace.

## Materials and methods

### Development of study materials

Two landrace accessions, Tuxpeño (PI 583894) from Sonora and Tabloncillo (PI 490927) from Nayarit, were acquired in 2012 from the National Plant Germplasm System (NPGS) of the United States Department of Agriculture (USDA). They were chosen as they represent two of 59 recognized races of Mexican landraces with diverse origin, phenotype, and utilization (Sanchez et al. 2000; Fernandez-Suarez et al. 2013).

These landraces were crossed with a maize inbred line that contained two GM traits, MON 89034 and MON 88017 developed by Monsanto Company (St. Louis, MO, USA). The MON 89034 transgene, produces two insecticidal proteins (Cry1A.105 and Cry2Ab2) that protect against feeding damage caused by several lepidopteran insect pests. Cry1A.105 is a modified *Bacillus thuringiensis* (*Bt*) Cry1A protein and Cry2Ab2 is a *Bt* (subsp. *kurstaki*) protein.

The MON 88017 transgene produces a modified *Bt* (subsp. *kumamotoensis*) Cry3Bb1 protein that protects against corn rootworm larval feeding. In addition, MON 88017 produces a protein (5-enolpyruvylshikimate-3-phosphate synthase) from *Agrobacterium* sp. strain CP4 (CP4 EPSPS), which confers tolerance to glyphosate, the active ingredient in the *Roundup*^®^^1^ family of agricultural herbicides.

The maize commercial product designated as VT3Pro, was developed by traditional breeding via crossing lines with MON 89034 and MON 88017. In this study, introgression of VT3Pro traits into the two landraces was conducted through traditional backcrossing and phenotypic selection in Puerto Rico, USA during the 2013–2015 period. Three backcross generations, using landraces as recurrent parents were completed to create materials with over 94% landrace genetics. During backcrossing, population size of each recurrent parent was on average 350 plants to maintain the genetic diversity of individuals within each landrace. The BC3 generation was sib-pollinated to produce sub-populations with and without the VT3Pro traits. During each cycle of backcrossing (BC1, BC2, and BC3), the presence of VT3Pro traits was ensured by herbicide application and molecular-based assays. During sib-pollination (BC3F2), the presence or absence of VT3Pro traits was determined by transgene zygosity testing. Progeny determined to be homozygous positive for both traits were sib-pollinated and the bulked seed was used as Tuxpeño VT3Pro and Tabloncillo VT3Pro entries in the field trials (Table 1). Similarly, progeny determined to be homozygous negative for both traits were sib-pollinated and bulked seed was used as Tuxpeño control and Tabloncillo control entries in the field trails (Table 1).

**Table 1:** Descriptive characteristics of landraces evaluated in 2015 and 2016 field trials

Landrace entries	Plant Inventory number^a^	Origin within Mexico	Kernel hardness^b^	Tassel color (%) (white:red)	Cob color (%) (white:red)	Kernel color (%) (white:with yellow:with red)^c^
Tuxpeño VT3Pro	PI 583894	Sonora	Intermediate	67.8:32.2	99.7:0.3	90.3:9.7:0.0
Tuxpeño control	PI 583894	Sonora	Intermediate	60.0:40.0	100.0:0.0	82.7:15.4:1.9
Tabloncillo VT3Pro	PI 490927	Nayarit	Intermediate	85.6:14.4	99.4:0.6	75.9:22.5:1.6
Tabloncillo control	PI 490927	Nayarit	Intermediate	81.1:18.9	100.0:0.0	76.5:21.9:1.6
Tuxpeño Sonora 51	PI 479072	Sonora	Intermediate	84.1:15.9	100.0:0.0	70.9:27.5:1.6
Tabloncillo Nayarit 63	PI 515340	Nayarit	Intermediate	84.1:15.9	98.4:1.6	61.9:32.5:5.6
Conico Norteño	PI 484473	Chihuahua	Hard	81.9:18.1	100.0:0.0	36.5:61.3:2.3
Harinoso 8	PI 490975	Sonora	Soft	85.6:14.4	85.3:14.7	73.4:25.3:1.3
Onaveño	PI 474210	Sonora	Intermediate	62.5:37.5	97.5:2.5	88.8:11.2:0.0
Blando/Blandito	PI 583893	Sinaloa	Very soft	28.8:71.2	84.0:16.0	88.0:0.0:12.0
Celaya 9	PI 484677	Guanajuato	Intermediate	71.9:28.1	100.0:0.0	99.4:0.0:0.6
Jala	PI 484692	Jalisco	Intermediate	68.8:31.2	85.0:15.0	91.2:7.5:1.3
Chalqueño	PI 485074	Puebla	Intermediate	88.1:11.9	93.6:6.4	53.9:39.0:7.1
Vandeño/Bandeño	PI 489509	Nayarit	Intermediate	58.8:41.2	100.0:0.0	3.7:95.0:1.3

^a^National Plant Germplasm System (NPGS)

^b^Kernel hardness per Vazquez-Carrillo et al. (2010)

^c^Proportion of ears with white kernels only, with yellow kernels (yellow or white/yellow), and with red kernels (red, red/white, red/yellow)

Additional landrace accessions, acquired in 2012 from the NPGS, were included in the field trials as reference materials to represent the range of variability typical for landraces grown in Mexico. The source seed for each reference entry used in this study was produced by sib-pollination in 2015 in the same nursery as the test and control materials. As genetic variability of Mexican landraces is associated mostly by region of their origin (Arteaga et al. 2016), these reference landraces were chosen to represent races from the Mexican states of Sonora, Chihuahua, Sinaloa, Nayarit, Jalisco, Guanajuato, and Puebla. The selected landrace references were: Tabloncillo Nayarit 63 (PI 515340), Tuxpeño Sonora 51 (PI 479072), Celaya 9 (PI 484677), Jala (PI 484692), Chalqueño (PI 485074), Vandeño/Bandeño (PI 489509), Onaveño (PI 474210), Blando/Blandito (PI 583893), Conico Norteño (PI 484473), and Harinoso 8 (PI 490975). Most of the reference landraces had intermediate kernel hardness but ranged from hard for Conico Norteño to soft for Harinoso 8 and very soft for Blando/Blandito, as per Vazquez-Carrillo et al. (2017) classification. Due to diversity of their kernel characteristics, these landraces have been used in traditional Mexican cuisine differently, such as for making tortillas (Tuxpeño, Tabloncillo, Celaya, Valndeño, and Onaveño), atoles (Blando and Harinoso 8), pinoles (Chalqueño, Jala, and Conico), botanas (Vandeño), or pozoles (Jala and Blando) (Fernandez-Suarez et al. 2013).

### Field observations

Field trials were grown in two USA states (Arizona and Texas) across 2 years (2015 and 2016). These four environments are designated as AZ-2015, TX-2015, AZ-2016, and TX-2016 (S-Table 1). The trials were planted either during the fall of 2015 or in the spring of 2016 representing two cultivation cycles with distinct growing conditions typical for maize farming systems utilized in Mexico (Louette and Smale 1998). The experiments were set in a randomized complete block design with four replications. Selected sites represented three distinct ecoregions (Madrean Archipelago, Sonoran Desert, and Western Gulf Coastal Plain) found in both southern USA and northern Mexico (Wiken et al. 2011). Furthermore, these field sites in southern USA regions were selected because they are congruent with regions (such as Baja California, Sonora, and Tamaulipas) where landraces, including Tuxpeño and Tabloncillo, are grown in Mexico (Lazos and Chauvet 2012).

Of a total of 14 entries evaluated in this study (Table 1), 10 were planted per site. Entries Tuxpeño VT3Pro, Tuxpeño control, Tabloncillo VT3Pro, Tabloncillo control, Tuxpeño Sonora 51, Tabloncillo Nayarit 63, Conico Norteño, and Harinoso 8 were planted at all sites. Onaveño and Blando/Blandito were planted at AZ-2016 only, Celaya 9 at AZ-2015 and TX-2016, Chalqueño at TX-2015 and TX-2016, Jala at AZ-2015, and Vandeño/Bandeño at TX-2015.

The entries were planted in eight-row plots that varied in size from 33.6 to 37.2 m^2^ depending on site. To ensure a uniform stand, plant thinning was conducted early in the season at all four sites. Agronomic practices (e.g., irrigation, fertilizers, pesticides) throughout the season were those typical to each region, and all maintenance operations were performed uniformly across plots. Protection against targeted pests was employed where necessary so that VT3Pro entries did not have advantage compared to control and reference landraces.

### Phenotypic characteristics

To assess the diversity of landraces in the study, three descriptive characteristics were evaluated including colors of tassels, cobs, and kernels (Table 1). Tassel color was evaluated during anthesis on 20 representative plants per replication and was defined as white or red (any shade). Color of the cobs and kernels were evaluated at harvest on 20 ears per replication. Cob color was either white or red. Ears were evaluated for kernel color as those with white kernels (all white), those with yellow kernels (all yellow or white and yellow), or ears with red kernels (all red, red and white, or red and yellow).

In addition to the three descriptive characteristics, a total of 22 phenotypic characteristics were evaluated to assess if there are differences between landraces with and without VT3Pro traits (S-Table 2). The evaluated characteristics included: early and final stand count, days to anthesis and silking, stay-green, ear and plant height, dropped ears, stalk and root lodging, test weight, grain moisture, 100-kernel weight, and yield. These characteristics are typically assessed by breeders, agronomists, and/or risk assessors for determining the potential impact of GM traits. Additional characteristics measured were: tassel length, number of tassel branches, ear length and diameter, kernel depth and number, cob diameter, and kernel rows per ear. They were assessed to better characterize the landraces used in this study and to include ear characteristics typically evaluated by Mexican farmers (Louette and Smale 1998). The evaluation was done using the inner rows of the eight-row plots.

### Responses to abiotic and biotic stressors

Plant responses to stressors (i.e., interactions between the crop plants and their receiving environment) were used to compare landraces with and without VT3Pro traits. The focus was on plant responses to abiotic stressors, arthropod damage, and disease damage. Entries were evaluated at all sites four times during the season (at V6–V8, V12-VT, R1–R3, and onset of R6). The stressors were selected based on current activity causing plant injury in the field trial or likelihood of that injury to occur in maize during a given observation period. Both active or anticipated stressors often varied among observations at a site, as well as among the evaluated sites.

Stressor observations were collected from each plot using a categorical scale of increasing severity of plant damage (*none*, *slight*, *moderate*, *severe*). A plot was given the assessment of *none* during the evaluation of a stressor if no symptoms were observed. An observation of *slight* was given if the symptoms were not damaging to plant development (e.g., minor feeding or lesions) so that likely no mitigation was required. If a plot was given the score of *moderate* for a stressor, this was considered as intermediate between *slight* and *severe*—a situation that would likely require mitigation. Finally, an observation of *severe* was given if the symptoms from a stressor were damaging to plant development (e.g., stunting or death) such that mitigation would unlikely be effective.

### Kernel compositional analysis

Kernel compositional analyses were conducted using harvested grain collected from each plot across the four tested environments. Grain samples were ground and homogenized prior to analyses that included proximate analytes, phenolic acids, and soluble carbohydrates. Proximate analytes were protein, total fat, ash, and carbohydrates (by calculation) following the methods described in Drury et al. (2008). Analyses were conducted at Covance Laboratories Inc. in Madison, WI, USA.

Phenolic acids were extracted from 50 mg samples of ground homogenized kernels by an improved microscale method as described in Zavala-López and García-Lara (2017). Extracts were analyzed by High-Performance Liquid Chromatography with Diode-Array Detection (HPLC–DAD) according to the method of Ayala-Soto et al. (2014) using an HPLC (Agilent 1100 Santa Clara, CA) coupled with a photodiode array (PDA) detector (Agilent G1315D, Santa Clara, CA). Linear gradient elution was performed with HPLC-grade water (acidified to pH 2 with trifluoroacetic acid) and acetonitrile, at a flow rate of 0.6 mL/min at 25 °C. Phenolic acids were separated on a Zorbax SB-Aq, 4.6 mm ID × 150 mm (3.5 µm) reverse phase column. Peak identification of trans-ferulic acid and *p*-coumaric acid was based on the retention times of authentic standards (Sigma-Aldrich, St. Louis, MO, USA).

Measured soluble carbohydrates were sucrose, fructose, and glucose. Samples of 50 mg of homogenized maize kernels were resuspended in 1 mL of distillated water and incubated at 25 °C for 15 min with constant agitation at 2500 rpm. Samples were then incubated overnight at 4 °C. An additional 0.5 mL of distilled water was added to all samples followed by incubation at 25 °C for 15 min with constant agitation at 2500 rpm. Samples were then centrifuged for 5 min at 6000 rpm, the supernatant was recovered and further centrifuged for 10 min at 13,000 rpm. The supernatant was recovered and filtered through a GHP 0.2 um filter (Pall Life Sciences, Ann Harbor, MI). Samples were analyzed within 24 hours.

Extracted sugars were analyzed by the method of Heredia-Olea et al. (2012) using an HPLC (Waters HPLC Breeze model, Milford, MA) equipped with a refractive index detector (Waters 2414 model, Milford, MA). The Empower software was used to process data and command the equipment. Sucrose, glucose, and fructose were separated using a Shodex SP0810 column (300 × 8.0 mm) with a flow rate of 0.6 mL/min of HPLC-grade water. The column, detector, and auto sampler temperatures were set at 80, 50, and 4 °C, respectively. Standards (Sigma-Aldrich, St. Louis, MO, USA) of sucrose, fructose and glucose, were used for peak identification and quantification. Analyses of phenolics and soluble carbohydrates were conducted at Tecnológico de Monterrey (Monterrey, México).

### Genetic segregation analysis

Segregation assessment of VT3Pro transgenes (MON 89034 and MON 88017) was conducted at the Monsanto research facilities in St. Louis, MO, USA. The backcrossed generations (BC1, BC2, BC3, and BC3F2) of Tuxpeño VT3Pro and Tabloncillo VT3Pro were evaluated to determine if MON 89034 and MON 88017 segregation follows Mendelian principles. Approximately 180 seeds of each generation per landrace were grown in a greenhouse. At early vegetative growth stage (V1–V4), leaf tissue from each seedling was sampled and gene presence was determined by event-specific polymerase chain reaction (PCR) analyses or by GeneCheck^®^ immunoassays (Cry1Ab QuickStix Lateral Flow test strips, Roundup Ready QuickStix Lateral Flow test strips—Envirologix Inc., Portland, ME, USA).

### Statistical analysis

#### Phenotypic and kernel compositional characterization

Descriptive characteristics were expressed as a proportion of individuals representing different colors of tassels, cobs, and kernels. The data was summarized using descriptive statistics. For other phenotypic and kernel compositional characteristics, the data was statistically analyzed using a linear mixed model under a randomized complete block design with landraces designated as a fixed effect. Sites, blocks nested within site, and site-by-landrace interactions were designated as random effects. Pairwise comparisons were made within the linear mixed model analysis to compare the test materials (Tuxpeño VT3Pro and Tabloncillo VT3Pro) to the corresponding conventional counterparts (Tuxpeño control and Tabloncillo control, respectively) across sites. These comparisons were also conducted across landraces in order to evaluate performance of landraces with and without VT3Pro traits across germplasm. Furthermore, pairwise comparisons were made between Tuxpeño control and Tabloncillo control to assess differences between the two landrace accessions. The level of significance was set at 5% (α = 0.05). Data was interpreted in a step-wise process similar to that described in Horak et al. (2015a).

#### Responses to abiotic and biotic stressors

The qualitative data regarding plant response to abiotic stressors, arthropod damage, and disease damage are categorical. The assessments of the landraces with and without VT3Pro traits were considered different in susceptibility or tolerance if the range of injury symptoms did not overlap between the compared entries across all four replications at a site (Horak et al. 2015a). Any observed differences were assessed for biological significance in the context of the range of the landrace reference materials, and for consistency across other observation times and sites. The responses to environmental stressors were also conducted across landraces in order to evaluate landraces with and without VT3Pro traits across germplasm.

#### Genetic segregation analysis

Chi square analysis was performed at a 5% level of significance to determine the segregation of the MON 88017 and MON 89034 genes for the four evaluated generations. The Chi square analysis was based on comparing the observed to the expected segregation ratio per Mendelian principles. Expected ratios for plants with and without transgenes for BC1, BC2, and BC3 generations was 1:1 for a single gene, or 1:1:1:1 when both genes are considered. Expected ratios for BC3F2 generations was 3:1 for a single gene, or 9:3:3:1 when both genes are considered. The Chi square analysis was completed for each landrace, as well as across landraces (which contributed to increased sample size).

## Results

Results from this study indicated similarity between landraces with and without VT3Pro traits, and diversity among reference and control landraces for the evaluated characteristics. The considered observations included: descriptive and phenotypic characteristics, plant responses to stressors, and kernel compositional characteristics.

### Descriptive characteristics

Tuxpeño with and without VT3Pro traits showed comparable values for the evaluated descriptive characteristics: proportion of plants with white (67.8% vs. 60%, respectively) and red tassels (32.2% vs. 40%), white cobs (99.7% vs. 100%), and white kernels (90.3% vs. 82.7%) (Table 1). Similarly, Tabloncillo with and without VT3Pro had comparable values for the evaluated descriptive characteristics, namely predominantly white tassels (85.6% vs. 81.1%), white cobs (99.4% vs. 100%), and white kernels (75.9% vs. 76.5%) (Table 1).

In contrast to similarities observed for landraces with and without VT3Pro traits, there were some differences between Tuxpeño and Tabloncillo landraces used as controls in this study such as the proportion of white tassels (60.0% vs. 81.1%, respectively), or proportion of ears with yellow kernels (15.4% vs. 21.9%, respectively) (Table 1). Diversity was also observed among landraces used as reference materials in this study. The proportion of plants with white tassels ranged from 88.1% for Chalqueño to as low as 28.8% for Blando/Blandito. Plants with white cobs ranged from 100% for Tuxpeño Sonora 51, Conico Norteño, Celaya 9, and Vandeño/Bandeño to 84.0% for Blando/Blandito. The proportion of white color kernels on an ear ranged from 99.4% for Celaya 9 to as low as 3.7% for Vandeño/Bandeño. Ears with yellow kernels ranged from 95.0% for Vandeño/Bandeño all the way to 0% for Blando/Blandito and Celaya 9, whereas ears with red kernels ranged from 12.0% for Blando/Blandito to 0% for Onaveño (Table 1).

### Phenotypic observations

The across-site analysis performed on 22 phenotypic characteristics indicated that there were no significant differences between landraces with and without VT3Pro traits for any of the evaluated characteristics except number of rows per ear (Table 2). However, the differences were less then the unit of measurement, thus considered too small to be biologically meaningful (13.7 vs. 13.2 for Tuxpeño and 11.8 vs. 11.7 across landraces).

**Table 2:** Mean value for phenotypic characteristics of landraces with and without VT3Pro traits in 2015 and 2016 field trials

Phenotypic characteristic (unit)	Tuxpeño		Tabloncillo		Across landraces		Reference range^a^
	VT3Pro	Control	VT3Pro	Control	VT3Pro	Control	
Early stand count	59.4	58.2	62.9	60.1	61.1	59.1	48.0–75.5
Days to anthesis^b^	74.5	72.6	68.3	68.0	71.4	70.3	57.8–107.3
Days to silking	77.6	75.3	70.7	70.9	74.2	73.1	57.5–117.8
Tassel length (cm)	37.7	36.7	36.7	36.1	37.2	36.6	32.5–42.3
Tassel branches/plant	15.3	14.6	14.1	13.3	14.7	13.9	8.8–19.9
Stay-green (1–9 scale)	5.6	6.1	7.0	6.1	6.3	6.3	2.0–8.0
Ear height (cm)^b^	124.1	122.1	110.3	110.4	117.2	116.3	96.6–205.8
Plant height (cm)^b^	217.1	216.1	194.4	201.0	205.9	208.6	181.5–281.9
Dropped ears^c^	0.1	0.1	0.1	0.1	0.1	0.1	0.0–0.5
Stalk lodged plants^b^	9.2	5.8	13.0	11.4	11.1	8.6	0.0–14.3
Root lodged plants	2.8	3.0	2.8	3.0	2.8	3.0	1.5–8.8
Final stand count	47.4	45.1	48.9	46.8	48.2	46.0	35.8–61.0
Ear length (cm)^b^	16.5	16.9	18.5	18.4	17.5	17.7	12.1–20.7
Ear diameter (cm)^b^	4.3	4.3	3.7	3.8	4.0	4.1	2.8–4.3
Cob diameter (cm)^b^	2.6	2.6	2.2	2.3	2.4	2.5	2.0–2.7
Kernel depth (mm)^b^	16.9	17.5	15.0	14.7	16.0	16.1	5.5–21.7
Rows/ear^b^	13.7*	13.2	9.8	10.2	11.8*	11.7	9.4–12.6
Kernels/ear^b^	365.9	371.9	280.5	275.4	323.2	323.7	41.4–338.1
100-kernel weight (g)	29.0	30.0	32.8	32.3	31.1	30.9	20.0–35.1
Grain moisture (%)	18.9	16.3	16.0	15.1	17.4	15.7	13.9–26.4
Test weight (kg/hl)	70.7	71.2	69.7	71.2	70.2	71.2	55.3–71.8
Yield (Mg/ha)	2.8	3.1	2.6	2.4	2.7	2.7	0.2–3.1

*Indicates statistically significant difference between VT3Pro landrace and the control (α = 0.05)

^a^Reference range is calculated from the minimum and maximum mean values among 10 unique reference materials

^b^Characteristics that showed statistically significant differences between Tuxpeño control and Tabloncillo control entries (α = 0.05)

^c^No statistical comparisons were made for dropped ears due to a lack of variability

In contrast to similarities observed between landraces with and without VT3Pro traits, there were significant differences detected between Tuxpeño and Tabloncillo landraces used as controls in this study for a number of characteristics, including days to anthesis (72.6 vs. 68.0), ear height (122.1 vs. 110.4 cm), plant height (216.1 vs. 201.0 cm), stalk lodging (5.8 vs. 11.4 plants), ear length (16.9 vs. 18.4 cm), ear diameter (4.3 vs. 3.8 cm), cob diameter (2.6 vs. 2.3 cm), kernel depth (17.5 vs. 14.7 mm), rows per ear (13.2 vs 10.2), and kernels per ear (371.9 vs. 275.4) (Table 2).

Furthermore, landraces used as references in this study have shown a broad range of values for many of the evaluated characteristics such as days to anthesis and silking, number of tassel branches, ear and plant height, kernel depth, rows per ear, kernels per ear, and yield (Table 2).

### Responses to abiotic and biotic stressors

The across-site stressor assessments included 139 observations (48 abiotic, 35 arthropod, and 56 disease) which did not result in any biologically meaningful differences between landraces with and without the VT3Pro traits (S-Table 3). For the abiotic stressors, no differences were observed between VT3Pro landraces and their respective controls for any of the comparisons except for Tabloncillo VT3Pro that showed lower wind damage compared to the control at the AZ-2015 site (slight vs. moderate rating, respectively). For arthropod stressors, no differences were observed between landraces with and without VT3Pro traits for any of the comparisons. Likewise, despite differences observed for gray leaf spot between landraces with and without VT3Pro traits at the AZ-2015 site (none vs. slight rating, respectively), no differences were observed for any of the other disease comparisons (S-Table 3). All VT3Pro landrace responses to the abiotic and biotic stressors were within the response range of the evaluated reference landraces (data not shown).

### Kernel composition

The across-site assessment for kernel composition included a total of 11 characteristics (four proximate analytes, four phenolics, and three soluble carbohydrates) (Table 3). The evaluation of proximate analytes encompasses major kernel components including protein, total fat, ash, and carbohydrates. In this study, no statistically significant differences were observed between landraces with and without VT3Pro traits for any evaluated proximate analyte (Table 3).

**Table 3:** Mean value for kernel compositional characteristics of landraces with and without VT3Pro traits in 2015 and 2016 field trials

Compositional characteristic (unit)	Tuxpeño		Tabloncillo		Across landraces		Reference range^a^
	VT3Pro	Control	VT3Pro	Control	VT3Pro	Control	
*Proximate analytes (% dw)*							
Protein	12.70	13.04	12.67	12.94	12.69	12.99	12.49–14.23
Total Fat	4.58	4.50	4.72	4.64	4.65	4.57	4.05–5.06
Ash	1.53	1.51	1.55	1.57	1.54	1.54	1.42–1.88
Carbohydrates (calculation)	81.21	80.97	81.07	80.85	81.14	80.91	79.01–81.65
*Phenolics (μg/g dw)*							
Ferulic acid (bound)^b,c^	1.99	1.93	1.49*	1.77	1.74*	1.85	1.15–2.53
Ferulic acid (free)	66.75	70.75	83.29	87.86	74.75	79.30	55.63–123.61
*p*-Coumaric acid (bound)	144.46	141.18	93.92	119.35	119.19	130.27	101.93–236.21
*p*-Coumaric acid (free)	8.07	7.10	7.30	6.61	7.69	6.85	0.50–20.23
*Soluble carbohydrates (% dw)*							
Sucrose	2.00	1.69	1.89	1.90	1.95	1.80	1.09–2.19
Fructose^b^	0.040*	0.029	0.038	0.044	0.039	0.037	0.037–0.073
Glucose	0.14	0.13	0.12	0.14	0.13	0.14	0.08–0.18

*Indicates statistically significant difference between VT3Pro landrace and the control (α = 0.05)

^a^Reference range is calculated from the minimum and maximum mean values among the unique reference materials

^b^Characteristics that showed statistically significant differences between Tuxpeño control and Tabloncillo control entries (α = 0.05)

^**c**^Expressed in mg/g dw

Major phenolic compounds in maize (*p*-coumaric and *trans*-ferulic acid) in their soluble and cell-wall bound forms were also analyzed. A statistically significant difference was observed for bound ferulic acid between Tabloncillo with and without VT3Pro traits. This difference (0.28 µg/g dw) is not biologically relevant when considering the much larger variability observed in the Tabloncillo control (0.76–3.10 µg/g dw) and that observed among the references (1.15–2.53 µg/g dw). Generally, the analyzed phenolic compounds showed a wide range of values among reference landraces, including free *p*-coumaric acid with up to 40-fold difference among the tested reference landraces (Table 3).

For the soluble carbohydrates analyzed (sucrose, glucose, and fructose) a statistically significant difference was observed for fructose between Tuxpeño with and without VT3Pro traits. The statistically significant difference is not relevant from a biological standpoint when considering the variation observed within the control (0.007–0.082% dw) and that observed among the references (0.037–0.073% dw) (Table 3). As expected, sucrose was the most abundant soluble sugar, followed by glucose and fructose.

### Genetic segregation analysis

Four segregating generations (BC1, BC2, BC3, and BC3F2) were analyzed using single (MON 89034, MON 88017) and two-gene (MON 89034 × MON 88017) models for VT3Pro landraces. The analysis was conducted for each landrace, as well as across the two landraces. None of the Chi square values were significantly different from expected in BC1 and BC3F2 generations (Table 4). Only one value deviated from the expected ratio in BC2 and BC3 generations each. However, when the analyses were done across landraces, which increased sample size, all the populations showed expected genetic ratios.

**Table 4:** Chi square segregation analyses of VT3Pro traits (MON 89034, MON 88017 and MON 89034 × MON 88017) across four generations of two maize landraces

Genes	BC1^a^	BC2^a^	BC3^a^	BC3F2^b^
MON 89034	1.31	0.16	4.83*	0.89
MON 88017	0.96	0.52	2.31	0.09
MON 89034 × MON 88017	2.69	1.00	7.60	0.85
*Tabloncillo*				
MON 89034	0.71	0.16	1.07	0.27
MON 88017	1.03	4.08*	1.91	0.12
MON 89034 x MON 88017	3.14	4.78	3.73	1.21
*Across landraces*				
MON 89034	0.06	0.32	0.72	0.21
MON 88017	0.00	3.75	0.01	0.00
MON 89034 × MON 88017	0.18	4.91	0.74	0.23

*Indicates statistically significant difference between observed and expected ratios using Chi square test statistic (*p* value < 0.05)

^a^Expected ratios for BC1, BC2, and BC3 generations was 1:1 for a single gene, or 1:1:1:1 for both genes combined

^b^Expected ratios for BC3F2 generations was 3:1 for a single gene, or 9:3:3:1 for both genes combined

## Discussion

### Diversity within and among landraces

The entries evaluated in this study were chosen to represent 10 out of the 59 recognized races of maize in Mexico (Sanchez et al. 2000). Observed characteristics provided evidence for the genetic diversity within and among landraces (Tables 1, 2, 3). For example, each landrace showed variation in tassel color ranging from white to different shades of red. This variation is not surprising as Mexican farmers do not typically select for plant characteristics such as tassel color (Louette and Smale 1998). Furthermore, evaluated landraces had plants with mostly white cobs, although a low percentage of red cobs was also observed for some populations. Kernel color showed the most variability, with ears ranging from a uniform kernel color (white, yellow, or red) to those with kernels of different colors. Some of this variation is due to cross-pollination by landraces grown in surrounding plots and xenia effect associated with this characteristic. However, most of the observed variability is due to diversity within landraces, considering that most kernels result from self- or sib-pollination even if grown in close proximity to a foreign pollen source (Baltazar et al. 2015).

These results confirm that the level of variation within a given landrace, very typical for Mexican open-pollinated maize populations (Sanchez et al. 2000), was maintained not only for reference landraces during seed increase, but also for the four entries developed during VT3Pro traits introgression (Tuxpeño VT3Pro, Tuxpeño control, Tabloncillo VT3Pro, and Tabloncillo control). Maintaining appropriate population size during backcrossing and selection along with employing sib-pollination for seed increase (mimicking open-pollination in farmers’ field) contributed to the retention of variation within each entry evaluated in this study.

Diversity was also observed among the landraces evaluated in this study. This is not surprising as they originated from different ecoregions associated with Mexican states of Sonora, Chihuahua, Sinaloa, Nayarit, Jalisco, Guanajuato, and Puebla. These seven states collectively represent one third of the total area of Mexico. The genetic diversity among these selected landraces is exemplified by descriptive characteristics like color of tassels, cobs, and kernels (Table 1), as well as phenotypic (Table 2), and compositional characteristics (Table 3). For example, wide ranges were observed for plant height (181.5–281.9 cm), ear length (12.1–20.7 cm), number of rows per ear (9.4–13.7), or *p*-coumaric acid (free) (0.50–20.23 mg/g dw) among the evaluated entries. Even landraces from the same region showed considerable diversity. For example, the proportion of plants with white tassels ranged from 62.5 to 85.6% for landraces that originated from Sonora, or from 58.8 to 85.6% for those from Nayarit. Similarly, the proportion of ears with white kernels ranged from 70.9 to 90.3% for entries from Sonora, or from 3.7 to 76.5% for those from Nayarit. This agrees with results reported in Pineda-Hidalgo et al. (2014) that showed high genetic diversity among maize landraces collected from a single state, even though the majority of the evaluated landraces were of either of Tuxpeño or Tabloncillo type.

**Fig. 1:**
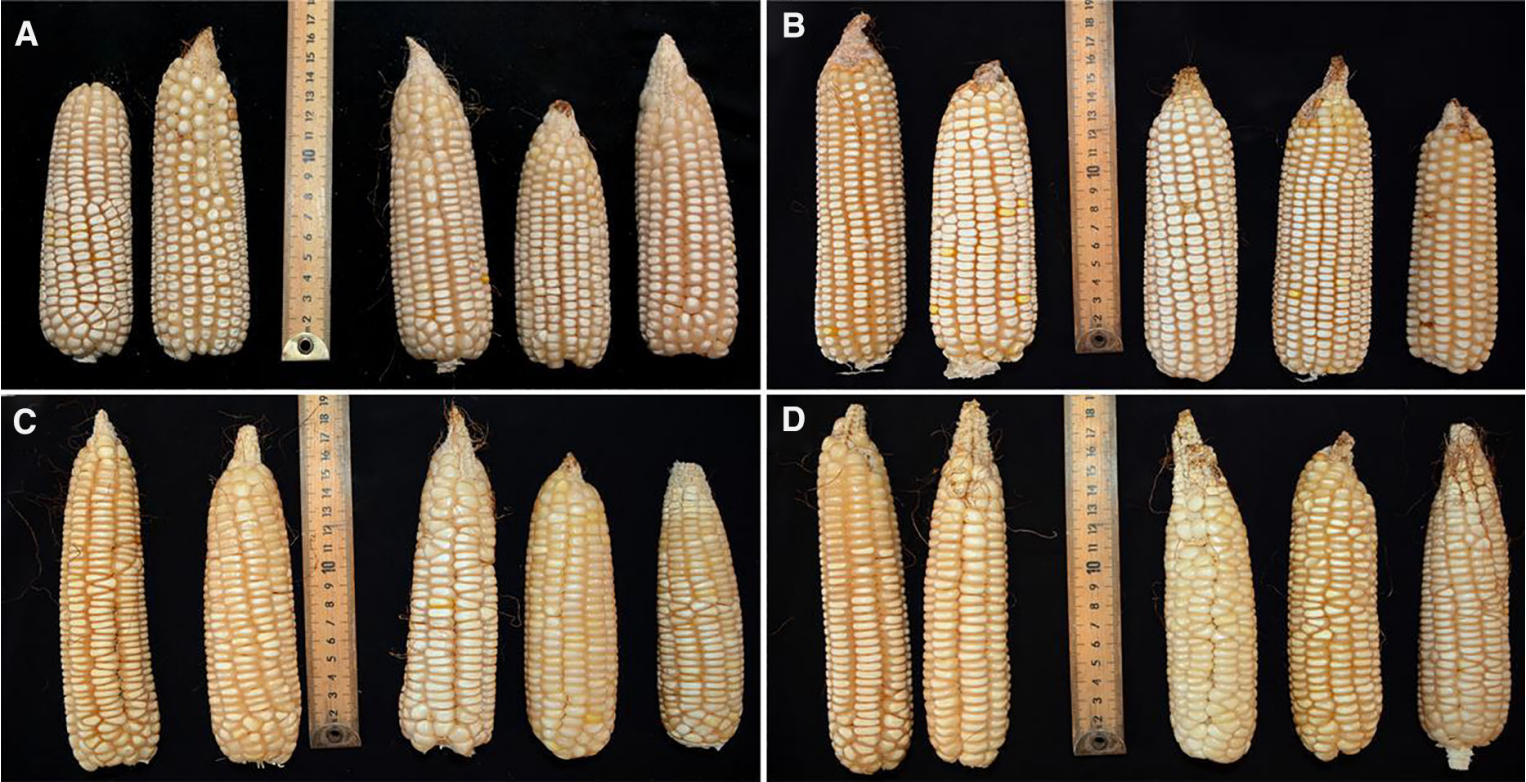
Harvested ears from Tuxpeño VT3Pro (**a**), Tuxpeño control (**b**), Tabloncillo VT3Pro (**c**), and Tabloncillo control (**d**)

The two landraces, Tuxpeño and Tabloncillo, which were used as controls in this study also showed differences in their performance, thus indicating that their identity and diversity was maintained through the process of material development for the field trials. Both landraces have predominantly white kernels, as this characteristic is generally preferred by Mexican farmers (Louette and Smale 1998), but the proportion of white kernels was higher for Tuxpeño (Table 1). Furthermore, Tabloncillo flowered a few days earlier, had shorter plants, longer ears, smaller ear and cob diameters, smaller kernel depth, and produced less kernels per ear compared to Tuxpeño (Table 2; Fig. 1). These results agree with the observations of Louette and Smale (1998) that landraces of Tabloncillo type are characterized by shorter plants, earlier maturity, and ears with fewer kernel rows. Similarly, Baltazar et al. (2005) showed that Tabloncillo flowered earlier compared to Tuxpeño.

### Landraces with and without VT3Pro

This study was undertaken to assess if the introduction of VT3Pro traits into landrace varieties exhibit changes in their characteristics beyond the intended traits (insect protection and herbicide tolerance). To our knowledge, this was the first study that evaluated maize landraces with introgressed biotechnology traits. The two landraces, Tuxpeño and Tabloncillo, were chosen because they are widely grown in Mexico (Sanchez et al. 2000; Lazos and Chauvet 2012), have broad representation on both coasts of Mexico, and are distributed in areas with modern maize hybrid production (Lazos and Chauvet 2012). Furthermore, they are of diverse origin, have different phenotypes, and are used for different food products (Sanchez et al. 2000; Vazquez-Carrillo et al. 2017).

The two introgressed biotechnology traits (MON 89034 and MON 88017) have been approved for cultivation in countries such as Argentina, Brazil, Canada, Colombia, Honduras, Paraguay, Philippines, South Africa, and the USA (ISAAA 2018). Prior to commercialization, hybrid maize containing MON 89034 and MON 88017 as single traits as well as stacked together were extensively tested for safety. The results of rigorous environmental risk and food safety assessments conducted for each trait have supported the conclusion that these products are as safe for human consumption and the environment as conventional varieties (Prado et al. 2014). These studies, along with years of commercial cultivation and use of these products, are evidence of the safe and effective use of GM maize. This study was conducted to test the hypothesis that Mexican maize landraces would remain unchanged in their characteristics, except for the associated GM traits, in the unlikely event of inadvertent introgression of VT3Pro transgenes.

Generally, no difference in performance was observed between landraces with and without VT3Pro traits indicating that the insertion of VT3Pro traits did not influence the phenotypic characteristics evaluated in this study. Similarly, Heredia-Diaz et al. (2016) showed no significant differences between maize hybrids with and without VT3Pro traits for early stand count, days to silking, days to anthesis, root lodging, stalk lodging, dropped ears, and final stand count. They did find few differences in performance, mostly due to pressure from target insect pests which resulted in better plant health of maize hybrids with VT3Pro traits. Graeber et al. (1999) concluded that incorporation of the Bt gene had little, if any, effect on agronomic performance of maize hybrids except for intended insect protection. Similar observations have been made for other transgenes, regardless of whether they were incorporated into maize (Nakai et al. 2015; Sammons et al. 2014) or other crops (Horak et al. 2007, 2015a).

Generally, no differences were observed between landraces with and without the VT3Pro traits in terms of responses to abiotic and biotic stressors that are not associated with intended traits (insect protection and herbicide tolerance). Similar results were observed by Ahmad et al. (2015) who showed comparable abundance and damage from non-target arthropods between maize hybrids with and without insect protection and herbicide tolerance GM traits. Other studies have also showed comparable responses to stressors between crops with and without GM traits (Horak et al. 2007, 2015b).

Tuxpeño is predominantly used for tortilla making, whereas Tabloncillo is primarily utilized for specialty products (Vazquez-Carrillo et al. 2017), which explains why the value of these landraces is closely associated with kernel compositional characteristics. The introgression of VT3Pro traits into Tuxpeño and Tabloncillo landraces did not result in unexpected changes in kernel composition associated with proximate analytes, carbohydrate metabolism, or phenolic content that may contribute to the characteristics of the grain and consequently impact the quality of food products. These results are supported by previous studies, which demonstrated that composition is affected more by environment and germplasm than by transgene insertion (Drury et al. 2008; McCann et al. 2007; Venkatesh et al. 2015).

Segregation analyses, conducted across multiple generations, indicated that VT3Pro transgenes segregated following Mendelian principles. Our results agree with observations that transgenes are expected to segregate the same as any endogenous gene (Bellon and Berthaud 2004).

In summary, this study was conducted to address questions regarding performance and inheritance of GM traits if inadvertently introgressed into maize landraces. These results indicate that Mexican landraces with and without VT3Pro traits have comparable phenotypic characteristics, responses to stressors, and kernel compositional characteristics with transgenes segregating the same as any endogenous gene. Our conclusions indicate that results obtained for maize hybrids can be extrapolated to maize landraces. These results should be taken into consideration when discussing benefits and risks associated with commercial production of GM maize hybrids in the centers of origin and diversity of maize.

## Electronic supplementary material

Below is the link to the electronic supplementary material.

Supplementary material 1 (DOCX 24 kb)

## Footnotes

1 ^®^*Roundup* is a registered trademark of Bayer Group.
