# Supplementary material for: Assessment of potential impacts associated with gene flow from transgenic hybrids to Mexican maize landraces
Source: Transgenic Res. 2019 Jun 27;28(5):509–23. doi: 10.1007/s11248-019-00160-3 (PMC6848245; doi:10.1007/s11248-019-00160-3)
Supplement: Supplementary file 1 — Supplementary material 1 (DOCX 24 kb) [file 11248_2019_160_MOESM1_ESM.docx]

**Supplemental Tables**

**S-Table 1** Details regarding field trials that evaluated maize landraces with and without VT3Pro traits

| Location | County, State | Ecoregion ^a^ | Planting Date | Harvest Date |
| --- | --- | --- | --- | --- |
| AZ-2015 | Graham, AZ | Madrean Archipelago | 07/24/2015 | 12/14/2015 |
| AZ-2016 | Pima, AZ | Sonoran Desert | 03/23/2016 | 09/01/2016 |
| TX-2015 | Waller, TX | Western Gulf Coastal Plain | 07/15/2015 | 11/20/2015 |
| TX-2016 | Waller, TX | Western Gulf Coastal Plain | 03/18/2016 | 08/10/2016 |

^a^ Ecoregions as described by Wiken et al. (2011)

**S-Table 2** Phenotypic characteristics evaluated for landraces with and without VT3Pro traits in 2015 and 2016 field trials

| Characteristic (units) | Evaluation Timing | Evaluation Description |
| --- | --- | --- |
| Early stand count | V2 – V5 | Number of emerged plants per two rows |
| Days to anthesis | VT | Days from planting until 50% anthesis |
| Days to silking | R1 | Days from planting until 50% silking |
| Tassel length (cm) | VT – R2 | Length from the base of the tassel to the tip of the main branch (20 plants) |
| Tassel branches/plant | VT – R2 | Total number of tassel branches (20 plants) |
| Stay-green | R5 - R6 | Visual rating on a 1-9 scale, where 1 = 90-100% and 9 = 0-19% green tissue |
| Ear height (cm) | R1 – R6 | Distance from the soil surface to the primary ear attachment node |
| Plant height (cm) | R1 – R6 | Distance from the soil surface to the flag leaf collar |
| Dropped ears | Pre-harvest | Number of dropped ears per two rows |
| Stalk lodged plants | Pre-harvest | Number of plants broken below the ear (per two rows) |
| Root lodged plants | Pre-harvest | Number of plants leaning more than 30° from vertical (per two rows) |
| Final stand count^3^ | Pre-harvest | Number of plants per two rows |
| Ear length (cm) | Harvest | Length of ears (20 plants) |
| Ear diameter (cm) | Harvest | Diameter of ears (20 plants) |
| Cob diameter (cm) | Harvest | Diameter of cobs (20 plants) |
| Kernel depth (mm) | Harvest | The difference between ear and cob diameter (20 plants) |
| Rows/ear | Harvest | Number of rows per ear (20 plants) |
| Kernels/ear | Harvest | Number of kernels per ear (20 plants) |
| 100-kernel weight (g) | Harvest | Weight of 100 kernels (20 plants) |
| Grain moisture (%) | Harvest | Moisture percentage of harvested shelled grain |
| Test weight (kg/hl) | Harvest | Test weight of harvested shelled grain |
| Yield (Mg/ha) | Harvest | Grain yield adjusted to 15.5% grain moisture |
|  |  |  |

**S-Table 3** Number of abiotic and biotic stressor comparisons where no differences were observed between the landraces with and without VT3Pro traits in 2015 and 2016 field trials

| Stressor Categories | Tuxpeño | Tabloncillo | Across Landraces | | |
| --- | --- | --- | --- | --- | --- |
| *Abiotic* | 48/48 | 47/48 | 48/48 | | |
| Drought | 7 (n) | 7 (n) | 7 | | |
| Frost | 2 (n-sl) | 2 (n-sl) | 2 | | |
| Hail | 3 (n-m) | 3 (n-m) | 3 | | |
| Heat | 7 (n-sl) | 7 (n-sl) | 7 | | |
| Mineral toxicity | 5 (n) | 5 (n) | 5 | | |
| Nutrient deficiency | 8 (n-m) | 8 (n-m) | 8 | | |
| Soil compaction | 1 (n) | 1 (n) | 1 | | |
| Sun scald | 4 (n-sl) | 4 (n-sl) | 4 | | |
| Wet soil/Flooding | 2 (n-sl) | 2 (n-sl) | 2 | | |
| Wind | 9 (n-se) | 8 (n-se)^a^ | 9 | | |
|  |  |  |  | | |
| *Arthropod* | 35/35 | 35/35 | 35/35 | | |
| Aphids (Aphididae) | 10 (n) | 10 (n) | 10 | | |
| Corn flea beetles (*Chaetocnema pulicaria*) | 1 (n) | 1 (n) | 1 | | |
| Corn rootworm beetles (*Diabrotica* spp.) | 2 (n) | 2 (n) | 2 | | |
| Grape colaspis (Chrysomelidae) | 1 (n) | 1 (n) | 1 | | |
| Grasshoppers (*Melanoplus* spp.) | 4 (n) | 4 (n) | 4 | | |
| June beetle (Scarabaeidae) | 1 (sl) | 1 (sl) | 1 | | |
| Leafhoppers (Cicadellidae) | 1 (n) | 1 (n) | 1 | | |
| Spider mites (*Tetranychus* spp.) | 8 (n-sl) | 8 (n-sl) | 8 | | |
| Stink bugs (Pentatomidae) | 5 (n-sl) | 5 (n-sl) | 5 | | |
| Thrips (Thysanoptera) | 2 (n) | 2 (n) | 2 | | |
|  |  |  |  | | |
| *Diseases* | 55/56 | 55/56 | 55/56 | | |
| Anthracnose | 7 (n) | 7 (n) | 7 | |  |
| Bacterial stalk rot of maize | 4 (n-se) | 4 (n-se) | 4 | |  |
| Corn stunt | 3 (n) | 3 (n) | 3 | |  |
| Downy mildew | 1 (n) | 1 (n) | 1 | |  |
| Ear rot | 4 (n-sl) | 4 (n-sl) | 4 | |  |
| Goss's bacterial wilt | 4 (n) | 4 (n) | 4 | |  |
| Gray leaf spot | 6 (n-sl)^b^ | 6 (n-sl)^b^ | 6^b^ | |  |
| Leaf blight (Northern and Southern) | 6 (n) | 6 (n-sl) | 6 | |  |
| Rust (Common and Southern) | 9 (n-m) | 9 (n-m) | 9 | |  |
| Common smut | 7 (n-sl) | 7 (n-sl) | 7 | |  |
| Stalk rot | 4 (n-se) | 4 (n-se) | 4 | |  |
|  |  |  | |  | |

Damage ratings = none (n), slight (sl), moderate (m), severe (se)

^a^ Eight of nine observations showed no difference between landrace with and without VT3Pro traits

^b^ Six of seven observations showed no difference between landraces with and without VT3Pro traits
